# Aspartate Aminotransferase – Risk Marker for Type-2 Diabetes Mellitus or Red Herring?

**DOI:** 10.3389/fendo.2014.00189

**Published:** 2014-11-04

**Authors:** Setor K. Kunutsor, Ali Abbasi, Tanefa A. Apekey

**Affiliations:** ^1^Cardiovascular Epidemiology Unit, Department of Public Health and Primary Care, University of Cambridge, Cambridge, UK; ^2^MRC Epidemiology Unit, Institute of Metabolic Science, Cambridge Biomedical Campus, University of Cambridge School of Clinical Medicine, Cambridge, UK; ^3^Department of Epidemiology, University Medical Center Groningen, University of Groningen, Groningen, Netherlands; ^4^Department of Internal Medicine, University Medical Center Groningen, University of Groningen, Groningen, Netherlands; ^5^Sport, Health and Nutrition Department, Leeds Trinity University, Leeds, UK

**Keywords:** aspartate aminotransferase, type-2 diabetes mellitus, liver enzymes, risk factors, meta-analysis

## Introduction

Gamma glutamyltransferase (GGT), alanine aminotransferase (ALT), aspartate aminotransferase (AST), and alkaline phosphatase (ALP) are common liver enzymes. Abnormal serum circulating levels of these enzymes may signal liver or cholestatic damage (1). Circulating GGT is present on the external surfaces of most cells, particularly hepatocytes, and is used as a biological marker of excessive alcohol intake (2). ALT and AST catalyze the transfer of amino groups to generate products in gluconeogenesis and amino acid metabolism (3, 4). Elevated serum levels of these aminotransferases signal acute or chronic liver injury (1). ALP is a hydrolase enzyme that transports metabolites across cell membranes, with elevated serum levels commonly used in clinical practice as a marker of liver or bony disease (1, 5).

In addition to their physiological functions, a growing body of evidence indicates that baseline serum levels of these enzymes may be associated with the development of a wide range of disease outcomes. Several reports have indicated that among these enzymes, elevated baseline levels of GGT and ALT are each associated with increased risk of future type-2 diabetes mellitus (T2DM) (6, 7). Indeed, the associations are apparent even within normal ranges of these enzymes. There is, however, considerable uncertainty regarding the association between AST level and risk of T2DM. In a recent review, we synthesized available prospective epidemiological data on the association between AST and incident T2DM (7). The pooled analyses involving 1,912 incident T2DM cases did not show a significant association between AST and risk of future T2DM. In contrast, a recently rigorously conducted prospective study involving 2,182 incident T2DM cases, reported a multivariate adjusted relative risk (RR) of 1.16 (1.02–1.31) for T2DM in a comparison of the highest to the lowest quartile of baseline AST levels (8). The association was continuous and extended well within the normal range of AST levels. This large study adds to the growing body of evidence that like GGT and ALT, elevated AST level may also be associated with increased risk for T2DM.

The prospective evidence on the association between AST and T2DM is inconclusive and this may be attributed to several reasons including: lack of adequate power, unmeasured confounding, or even over-adjustment for potential intermediates by previous studies. Given that levels of serum liver enzymes (GGT, ALT, and AST) (i) are strongly environmentally and genetically correlated with one another (9), and (ii) have shared genetic variances (10), the evidence is suggestive of common biological pathways affecting levels of these enzymes. There is therefore a possibility that the association between AST level and risk of T2DM might be mediated through the effects of the other liver enzymes. The potential impact of other liver enzymes on the AST–T2DM association is unclear as it is uncertain whether adjusting for such putative intermediates is appropriate. There are indications that further adjustment for other liver enzymes may be responsible for the substantially attenuated or null associations observed in several studies. To help clarify the evidence, we report an updated review.

## Methods

We searched MEDLINE, EMBASE, and Web of Science electronic databases for published studies reporting on the associations between baseline AST levels and incident T2DM since the date of the previous review (7). Details of the review methodology and inclusion/exclusion criteria have been reported previously (7). Briefly, studies were only included if they had at least 1 year of follow-up, recruited participants from approximately general populations (i.e., did not select participants on the basis of pre-existing diabetes or known liver disease), and excluded participants with marked elevations in AST levels. Data were abstracted on several study characteristics including degree of adjustment for potential confounders (defined as “+” when RRs were adjusted for age and/or sex; “++” further adjustment for established diabetes risk factors; and “+++” additional adjustment for liver enzymes). We separately extracted estimates from models adjusting for established and potential diabetes risk factors and models with additional adjustment for other liver enzymes. We contacted authors of eligible studies where the published data were insufficient, to provide relevant missing information. Risk estimates were transformed to involve comparisons between the top third and bottom third of the study population’s baseline distribution of AST levels. Consistency of findings across studies was assessed by standard χ^2^ and *I*^2^ statistics (11). Prespecified sources of potential heterogeneity were explored by using random-effects meta-regression. Evidence of publication bias was assessed using Begg’s funnel plots and Egger’s asymmetry test (12, 13). All analyses were performed using Stata release 12 (StataCorp, College Station, TX, USA).

## Results

**Figure 1:**
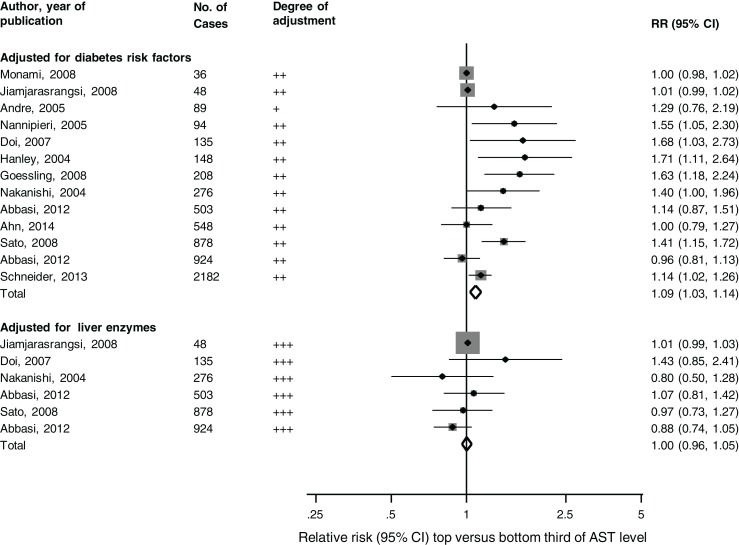
**Prospective studies of aspartate aminotransferase levels and type-2 diabetes mellitus risk, grouped according to covariate adjustment levels**. The summary estimates presented were calculated using random-effects models; degree of adjustment: +, adjusted for age and/sex; ++, additionally adjusted for diabetes risk factors; +++, further adjusted for other liver enzymes; AST, aspartate aminotransferase; bars, 95% CI; RR, relative risk.

Data were available on 90,975 participants from 13 unique prospective cohort studies (14–24) (of which, 9 were included in the previous review) (Table S1 in Supplementary Material). The cumulative analysis involved 6,069 incident T2DM cases, collected over average follow-up periods ranging from 3 to 20 years. The pooled random-effects RR (95% CI) for T2DM in a comparison of extreme thirds of AST level was 1.09 (1.03–1.14) in studies that adjusted for potential diabetes risk factors. The corresponding RR was 1.00 (0.96–1.05) in studies that additionally adjusted for other liver enzymes (*P* for meta-regression = 0.05; Figure 1). There was substantial heterogeneity among the studies that adjusted for diabetes risk factors (*I*^2^ = 73%: 54–85%, *P* < 0.001). The inconsistency was to a large part explained by duration of follow-up (*P* for meta-regression = 0.004), with combined estimates from studies with follow-up duration ≥5 years (1.28, 95% CI: 1.11–1.49) being stronger than those with shorter follow-up duration (1.02, 95% CI: 0.98–1.05). Egger’s test was not significant (*P* = 0.92), consistent with observed funnel plot symmetry.

## Discussion

The pattern of findings from the available evidence suggest that elevated AST is associated with increased risk of T2DM after controlling for potential confounding. Stronger associations were demonstrated for studies with longer duration of follow-up, findings, which confirm the speculative suggestion that duration of follow-up plays a role in the association between liver aminotransferases and T2DM (7). This finding may, however, require replication in further studies. The association of AST with T2DM was, however, abrogated by further adjustment for other liver enzymes. These results must be interpreted with caution given potential limitations such as effects of residual confounding, variably adjusted data, and the possibility for bias when attempting to assess the impact of putative intermediates (25) such as other liver enzymes (GGT and ALT). If other liver enzymes mediate the association, then correction for these enzymes may be an over-adjustment by the studies concerned. The relationship between these liver enzymes and T2DM may be more complex than generally appreciated. Future studies are warranted to help provide insight into the nature of these processes and determine the joint role of these liver enzymes in the pathophysiology of T2DM.

## Conflict of Interest Statement

The authors declare that the research was conducted in the absence of any commercial or financial relationships that could be construed as a potential conflict of interest.

## Supplementary Material

The Supplementary Material for this article can be found online at http://www.frontiersin.org/Journal/10.3389/fendo.2014.00189/full

## References

[1] Giannini EG Testa R Savarino V. Liver enzyme alteration: a guide for clinicians. CMAJ (2005) 172:367–79. 10.1503/cmaj.1040752 15684121 PMC545762

[2] Whitfield JB. Gamma glutamyl transferase. Crit Rev Clin Lab Sci (2001) 38:263–355. 10.1080/20014091084227 11563810

[3] Wroblewski F Ladue JS. Serum glutamic pyruvic transaminase in cardiac with hepatic disease. Proc Soc Exp Biol Med (1956) 91:569–71. 10.3181/00379727-91-22330 13323003

[4] Wroblewski F Ladue JS. Serum glutamic oxalacetic aminopherase (transaminase) in hepatitis. J Am Med Assoc (1956) 160:1130–4. 10.1001/jama.1956.02960480030008a 13295080

[5] Tonelli M Curhan G Pfeffer M Sacks F Thadhani R Melamed ML Relation between alkaline phosphatase, serum phosphate, and all-cause or cardiovascular mortality. Circulation (2009) 120:1784–92. 10.1161/CIRCULATIONAHA.109.851873 19841303

[6] Fraser A Harris R Sattar N Ebrahim S Davey Smith G Lawlor DA. Alanine aminotransferase, gamma-glutamyltransferase, and incident diabetes: the British women’s heart and health study and meta-analysis. Diabetes Care (2009) 32:741–50. 10.2337/dc08-1870 19131466 PMC2660465

[7] Kunutsor SK Apekey TA Walley J. Liver aminotransferases and risk of incident type 2 diabetes: a systematic review and meta-analysis. Am J Epidemiol (2013) 178:159–71. 10.1093/aje/kws469 23729682

[8] Schneider AL Lazo M Ndumele CE Pankow JS Coresh J Clark JM Liver enzymes, race, gender and diabetes risk: the atherosclerosis risk in communities (ARIC) study. Diabet Med (2013) 30:926–33. 10.1111/dme.12187 23510198 PMC3715563

[9] Whitfield JB Zhu G Nestler JE Heath AC Martin NG. Genetic covariation between serum gamma-glutamyltransferase activity and cardiovascular risk factors. Clin Chem (2002) 48:1426–31. 12194918

[10] Rahmioglu N Andrew T Cherkas L Surdulescu G Swaminathan R Spector T Epidemiology and genetic epidemiology of the liver function test proteins. PLoS One (2009) 4:e4435. 10.1371/journal.pone.0004435 19209234 PMC2636884

[11] Higgins JP Thompson SG. Quantifying heterogeneity in a meta-analysis. Stat Med (2002) 21:1539–58. 10.1002/sim.1186 12111919

[12] Begg CB Mazumdar M. Operating characteristics of a rank correlation test for publication bias. Biometrics (1994) 50:1088–101. 10.2307/2533446 7786990

[13] Egger M Davey Smith G Schneider M Minder C. Bias in meta-analysis detected by a simple, graphical test. BMJ (1997) 315:629–34. 10.1136/bmj.315.7109.629 9310563 PMC2127453

[14] André P Balkau B Born C Royer B Wilpart E Charles MA Hepatic markers and development of type 2 diabetes in middle aged men and women: a three-year follow-up study. The D.E.S.I.R. study (data from an epidemiological study on the insulin resistance syndrome). Diabetes Metab (2005) 31:542–50. 10.1016/S1262-3636(07)70229-X 16357802

[15] Abbasi A Bakker SJ Corpeleijn E van derADL Gansevoort RT Gans RO Liver function tests and risk prediction of incident type 2 diabetes: evaluation in two independent cohorts. PLoS One (2012) 7:e51496. 10.1371/journal.pone.0051496 23284703 PMC3524238

[16] Goessling W Massaro JM Vasan RS D’Agostino RB Sr Ellison RC Fox CS. Aminotransferase levels and 20-year risk of metabolic syndrome, diabetes, and cardiovascular disease. Gastroenterology (2008) 135(1935–44):44e1. 10.1053/j.gastro.2008.09.018 19010326 PMC3039001

[17] Monami M Bardini G Lamanna C Pala L Cresci B Francesconi P Liver enzymes and risk of diabetes and cardiovascular disease: results of the firenze bagno a ripoli (FIBAR) study. Metabolism (2008) 57:387–92. 10.1016/j.metabol.2007.10.015 18249212

[18] Doi Y Kubo M Yonemoto K Ninomiya T Iwase M Tanizaki Y Liver enzymes as a predictor for incident diabetes in a Japanese population: the Hisayama study. Obesity (Silver Spring) (2007) 15:1841–50. 10.1038/oby.2007.218 17636103

[19] Hanley AJ Williams K Festa A Wagenknecht LE D’Agostino RB Jr Kempf J Elevations in markers of liver injury and risk of type 2 diabetes: the insulin resistance atherosclerosis study. Diabetes (2004) 53:2623–32. 10.2337/diabetes.53.10.2623 15448093

[20] Sato KK Hayashi T Nakamura Y Harita N Yoneda T Endo G Liver enzymes compared with alcohol consumption in predicting the risk of type 2 diabetes: the Kansai healthcare study. Diabetes Care (2008) 31:1230–6. 10.2337/dc07-2184 18316395

[21] Nannipieri M Gonzales C Baldi S Posadas R Williams K Haffner SM Liver enzymes, the metabolic syndrome, and incident diabetes: the Mexico city diabetes study. Diabetes Care (2005) 28:1757–62. 10.2337/diacare.28.7.1757 15983331

[22] Nakanishi N Suzuki K Tatara K. Serum gamma-glutamyltransferase and risk of metabolic syndrome and type 2 diabetes in middle-aged Japanese men. Diabetes Care (2004) 27:1427–32. 10.2337/diacare.27.6.1427 15161799

[23] Jiamjarasrangsi W Sangwatanaroj S Lohsoonthorn V Lertmaharit S. Increased alanine aminotransferase level and future risk of type 2 diabetes and impaired fasting glucose among the employees in a university hospital in Thailand. Diabetes Metab (2008) 34:283–9. 10.1016/j.diabet.2008.01.009 18486512

[24] Ahn HR Shin MH Nam HS Park KS Lee YH Jeong SK The association between liver enzymes and risk of type 2 diabetes: the Namwon study. Diabetol Metab Syndr (2014) 6:14. 10.1186/1758-5996-6-14 24502834 PMC3918101

[25] Robins JM Greenland S. Identifiability and exchangeability for direct and indirect effects. Epidemiology (1992) 3:143–55. 10.1097/00001648-199203000-00013 1576220

