# Supplementary material for: Aspartate Aminotransferase – Risk Marker for Type-2 Diabetes Mellitus or Red Herring?
Source: Front Endocrinol (Lausanne). 2014 Nov 4;5:189. doi: 10.3389/fendo.2014.00189 (PMC4219379; doi:10.3389/fendo.2014.00189)
Supplement: Supplementary file 1 [file Table_1.PDF]

Supplementary Table - Characteristics of published prospective studies evaluating associations between aspartate aminotransferase and incident Type 2 diabetes mellitus

First Author, Year	Name of study/Source of participants	Location	Year of baseline survey	Baseline age range (yrs)	% male	Follow up years	Total participants	No. of cases	Covariates adjusted for
Hanley, 2004	IRAS	US	1992-94	40-69	74	5	906	148	Age, sex, ethnicity, clinical center, alcohol, smoking, WC, TG, HDL, IGT, $S_{i,}$ AIR
Nakanishi, 2004	Office Workers	Japan	1994	35-59	100	7	3,260	276	Age, FHxD, BMI, alcohol, smoking, PA, FPG, WBC count, other liver enzymes
Andre, 2005	DESIR	France	1994-96	30-65	49	3	4,201	89	Age
Nannipieri, 2005	MCDS	Mexico	1987-92	35-64	40	7	1,233	94	Age, BMI, WC, fasting insulin, alcohol
Doi, 2007	Hisayama	Japan	1988	40-79	40	9	1,804	135	Age, FHxD, fasting insulin, BMI, WHR, TC, HDL-C, TGs, CRP, HPT, alcohol, smoking, PA, other liver enzymes
Goessling, 2008	FHS Offspring	US	1978-82	44*	44	20	2,138	208	Age, sex, smoking, menopause, alcohol, BMI, glucose, interim weight change
Jiamjarasrangsi, 2008	University Hospital	Thailand	2001-05	35-60	19	3	2,370	48	Age, sex, education, alcohol, smoking, FHxD, BMI, SBP, DBP, cholesterol, TG, FPG, BUN, uri acid, follow-up time, ALT
Monami, 2008	FIBAR	Italy	2001-03	40-75	43	3	2,662	36	Age, sex, alcohol, smoking, FPG
Sato, 2008	KHS	Japan	2000-01	40-55	100	4	8,576	878	Age, BMI, FPG, smoking, parental hx of diabetes, walk to work, regular leisure-time PA, alcohol consumption, FPG, other liver enzymes
Abbasi, 2012	EPIC-NL	The Netherlands	1993-1997	20-70	25.7	10.2	38,379	924	Age, sex, BMI, smoking, parental hx of diabetes, hypertension, glucose, uric acid, GGT
Abbasi, 2012	PREVEND	The Netherlands	1997-1998	28-75	49.1	7.7	7,952	503	Age, sex, BMI, smoking, parental hx of diabetes, hypertension, glucose, uric acid, GGT
Schneider, 2013	ARIC	US	1996-1998	62.7*	42.7	12	9,337	2,182	Age, gender, race, education, family income, smoking, alcohol consumption, BMI, WC, TGs, HDL-C, SBP, DBP, use of HPT medications, C-RP

Ahn, 2014	Namwon	Korea	2004-2007	45-74	39.9	4.2	8,157	548	Age, BMI, WC, TC, HDL-C, TGs, alcohol intake, smoking status, PA, follow-up period, CRP, fasting glucose, HOMA
**Total**							**90,975**	**6,069**	

*Mean age at baseline; DESIR, Data from Epidemiological Study on the Insulin Resistance Syndrome; EPIC, European Prospective Investigation into Cancer; FHS, Framingham Heart Study; FIBAR, Firenze Bagno a Ripoli; Insulin Resistance Atherosclerosis Study; KHS, Kansai Healthcare Study; MCDS, Mexico City Diabetes Study; NL, Netherlands; PREVEND, Prevention of Renal and Vascular End-stage Disease

AIR, acute insulin response; BMI, body masss index; BUN, blood urea nitrogen; DBP, diastolic blood pressure; FHxD, family history of diabetes; FPG, fasting plasma glucose; FSG, fasting serum glucose; GGT, gamma glutamyltransferase; HDL-C, high density lipoprotein cholesterol; CRP, C-reactive protein; HOMA-IR, homeostasis model assessment of insulin resistance; HPT, hypertension; PA, physical activity; S_i, insulin sensitivity index; SBP, systolic blood pressure; TC, total cholesterol; TG, triglycerides; WC, waist circumference; WHR, waist-to-hip ratio.
